# WHAT DOES IT MEAN TO REST? A STUDY OF CAREGIVERS CARING FOR A PERSON WITH DEMENTIA

**DOI:** 10.1093/geroni/igad104.0468

**Published:** 2023-12-21

**Authors:** Sarah Hahn

**Affiliations:** Mercy College, Dobbs Ferry, New York, United States

## Abstract

The number of informal caregivers caring for a person with dementia (PWD) has been increasing steadily over the past decade; more than 11 million Americans provide unpaid care as of 2022 (Alzheimer’s Association, 2022). The impact of caregiving is immense; caregivers risk having chronic health issues, poor emotional well-being, and physiological changes (Alzheimer’s Association, 2022). Respite care provides a temporary break for the caregiver, but is often underutilized (O’ Shea, et al., 2019). Research has found that the responsibilities of caregiving for a PWD, specifically the mental load, is a 24-hour, 7-day position that leaves little room for rest (Chew, et al., 2022). However, while there are several definitions of what rest and respite is in the gerontological literature (O’ Shea, et al., 2019), not much is known on the experiences and achievement of rest by caregivers of PWD. This study explored the concept of rest to caregivers of PWD by using phenomenological interviews to understand the meanings attributed to it. Findings indicated three superordinate themes: “roadblocks”, “the actuality”, and “the ideal”, as well as subsequent subthemes.

